# Exosome-mediated miR-7-5p delivery enhances the anticancer effect of Everolimus via blocking MNK/eIF4E axis in non-small cell lung cancer

**DOI:** 10.1038/s41419-022-04565-7

**Published:** 2022-02-08

**Authors:** Sile Liu, Weiyuan Wang, Yue Ning, Hongmei Zheng, Yuting Zhan, Haihua Wang, Yang Yang, Jiadi Luo, Qiuyuan Wen, Hongjing Zang, Jinwu Peng, Jian Ma, Songqing Fan

**Affiliations:** 1 Department of Pathology, The Second Xiangya Hospital, Central South University, Changsha, Hunan China; 2 Department of Pathology, Xiangya Hospital, Central South University, Changsha, Hunan China; 3 Cancer Research Institute, Central South University, Changsha, Hunan China

**Keywords:** Drug development, Growth factor signalling, Oncogenesis

## Abstract

Everolimus is a kind of mammalian target of rapamycin (mTOR) inhibitors. Activated mitogen-activated protein kinase interacting kinases/eukaryotic translation initiation factor 4E (MNK/eIF4E) axis plays a crucial role in resistance to Everolimus in non-small cell lung cancer (NSCLC). The eIF4E phosphorylation increased by mTOR inhibitors is mainly mediated by MNKs. However, the mechanisms are poorly understood. Recently, extensive reprogramming of miRNA profiles has also been found after long-term mTOR inhibitor exposure. Our previous studies have confirmed that tumor suppressor miR-7-5p is decreased in A549 cells after treatment with Everolimus. Exactly, MNK1 is the target of miR-7-5p. In this study, we investigated the biological functions and potential molecular mechanisms of miR-7-5p in the NSCLC undergoing treatment with Everolimus. We confirmed that Everolimus targeted mTORC1 inducing NSCLC cells to secrete miR-7-5p-loaded exosomes in Rab27A and Rab27B-dependent manners. Loss of intracellular miR-7-5p induced phosphorylation of MNK/eIF4E axis, but a supplement of extra exosomal miR-7-5p could reverse it. Of note, both low expression of miR-7-5p and elevated MNK1 protein were associated with a poor prognosis of NSCLC. Both endogenous miR-7-5p and exo-miR-7-5p enhanced the therapeutic efficacy of Everolimus by inhibiting the proliferation, migration, and metastasis of NSCLC in vitro and in vivo. The combination of miR-7-5p with Everolimus induced apoptosis to exhibit a synergistic anticancer therapeutic efficacy through dual abrogation of MNK/eIF4E and mTOR in NSCLC. In conclusion, Everolimus decreases the intracellular miR-7-5p by releasing of miR-7-5p loaded exosomes from NSCLC cells in Rab27A and Rab27B dependent manners. Either endogenous miR-7-5p or exo-miR-7-5p combined with Everolimus can enhance the anticancer efficacy by targeting MNK/eIF4E axis and mTOR. Besides, both low levels of miR-7-5p and positive expression of MNK1 act as independent poor prognostic biomarkers for NSCLC. Therefore, restoring miR-7-5p carried by exosome may be a promising novel combined therapeutic strategy with Everolimus for NSCLC.

## Introduction

The mammalian target of rapamycin (mTOR) is a serine/threonine kinase that can regulate cell growth and proliferation under physiological and pathological conditions. Dysregulation of the mTOR signaling pathway can be seen in many cancers, including non-small cell lung cancer (NSCLC). Furthermore, abnormal activation of the PI3K-AKT-mTOR pathway has been proved to generate acquired resistance to epidermal growth factor receptor (EGFR) tyrosine kinase inhibitors (TKIs) in NSCLC [1]. The functions of mTOR are mainly exercised by forming two different complexes, named mTOR complex 1 (mTORC1) and mTOR complex 2 (mTORC2) [2]. The combination of mTORC1 inhibitor Everolimus with EGFR-TKIs has clinical efficacy in TKIs-resistant NSCLC cell lines [3], but the subsequent clinical trials show limited effect of combination therapy on unselected NSCLC patients [4]. Everolimus treatment causes genomic instability, leading to secondary resistance [5]. Activation of other regulatory proteins or survival cascades are also involved in resistance to Everolimus, like mitogen-activated protein kinase-interacting kinases 1 and 2 (MNK1 and MNK2). The activated MNKs continuously phosphorylate eIF4E, and play a crucial role in mediating resistance to rapamycin in NSCLC [6]. Moreover, eIF4E phosphorylation increased by mTOR inhibitors is mainly mediated by MNKs [7]. However, the mechanisms about phosphorylation of MNK/eIF4E axis induced by Everolimus are poorly understood.

Long-term treatment with mTOR inhibitor rapamycin showed extensive reprogramming of miRNA profiles, characterized by down-regulation of tumor suppressor miRNAs. However, the delivery of tumor suppressor miRNAs would restore the sensitivity to rapamycin [8]. Our previous studies have confirmed that Everolimus activates the feedback of the MNK/eIF4E axis in NSCLC cells. Besides, there are changes of the miRNA profiles in A549 cells with Everolimus treatment [9]. MIR-7 is regarded as a tumor suppressor in NSCLC, which dominantly regulates several basic cellular processes including proliferation, differentiation, apoptosis, migration and expression of stem cell features [10]. Also miR-7 can decrease EGFR mRNA level [11] and regulate many genes of the mTOR pathway, like MNK, eIF4E and 70 kDa ribosomal protein S6 kinase (p70S6K) [12]. MIR-7, as a specific biotherapeutic agent in NSCLC, needs to be explored. Therefore, the down-regulated miR-7-5p has attracted our attention.

Exosomes derived from tumor cells carry the components of membranes, and cells fall off into the extracellular space and cause the loss of intracellular proteins, miRNAs, and lncRNAs through the secretion. Therefore, exosomes are important for the early diagnosis of disease, assessment of the pathogen burden and monitoring of the response to treatment [13]. This secretion-dependent mechanism is regulated by mTORC1 [14]. The occurrence of resistance mechanism of Everolimus might be related to the release of exosomes.

This study investigated whether Everolimus inhibits mTORC1 by downregulating miR-7-5p in an exosome-dependent manner and its mechanisms in NSCLC. We found that Everolimus promoted the secretion of miR-7-5p loaded exosomes derived from tumor cells, decreasing intracellular miR-7-5p and activating the MNK/eIF4E axis. Hopefully, either endogenous miR-7-5p or exo-miR-7-5p combined with Everolimus synergistically enhances the anticancer efficacy through targeting MNK/eIF4E axis and mTOR.

## Results

### Everolimus targeted mTORC1 inducing NSCLC cells to secrete miR-7-5p-loaded exosomes in Rab27A and Rab27B dependent manners

**Fig. 1:**
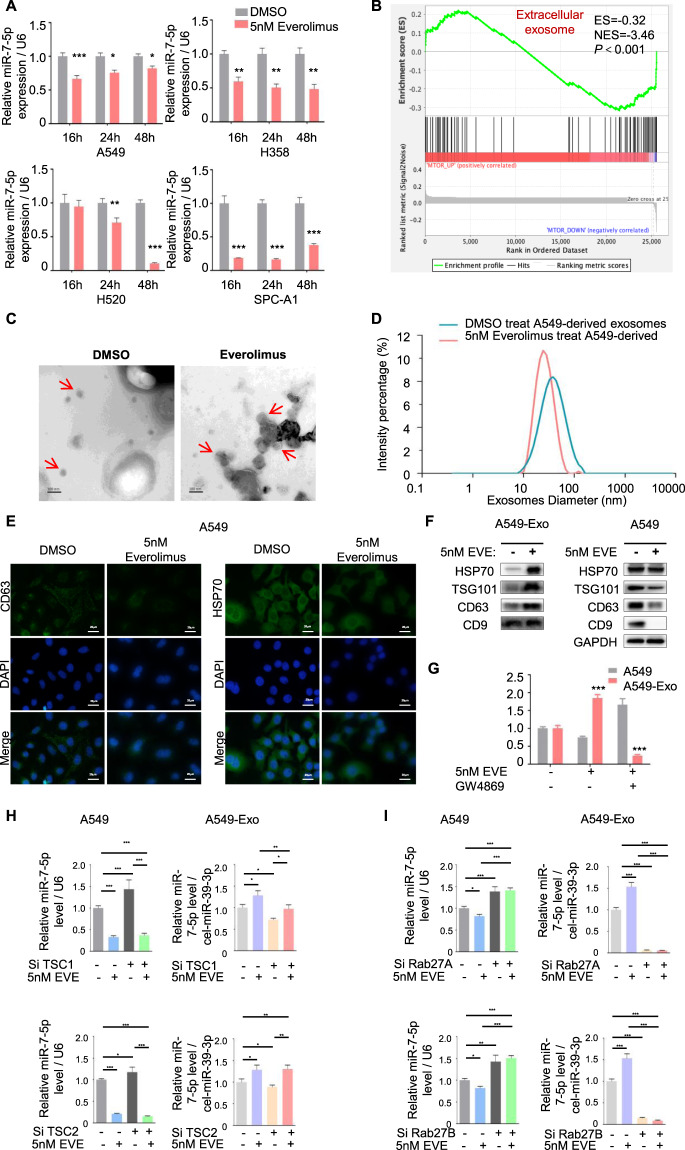
Everolimus targeted mTORC1 inducing NSCLC cells to secrete miR-7-5p-loaded exosomes in Rab27A and Rab27B dependent manners. **A** The levels of miR-7-5p in NSCLC cells (A549, H358, H520 and SPC-A1) treated with Everolimus were measured by qPCR for 16 h, 24 h and 48 h. *T*-test. Each bar represents mean ± SD. **B** GSEA analysis showed the difference gene set between mTOR^low^ and mTOR^high^. ES, enrichment score; NES, normalized enrichment score. **C** Transmission electron microscopy images for exosomes pointed by the red arrow (scale bar=100 nm). **D** Size distribution analysis of purified exosomes by Zetasizer Nano ZS90. **E** Immunofluorescence was carried out to detect the expression of exosomal marker CD63 and HSP70 (green) in A549 cell line with or without Everolimus treatment. **F** Exosomal markers including HSP70, TSG101, CD63 and CD9 were analyzed by Western blotting in A549 cells and A549-derived exosomes. DAPI (blue) was used for nuclear staining. **G** The levels of intracellular and exosomal miR-7-5p were measured by qPCR. A549 cells were treated with 5 nM Everolimus combined with or without 10 μM GW4869 for 24 h. **H** A549 cells were transfected with siTSC1/2 and treated with or without Everolimus for 24 h. The qPCR detected the levels of intracellular and exosomal miR-7-5p in the corresponding cells above. **I** A549 cells were transfected with siRab27A/B and treated with or without Everolimus for 24 h. The qPCR detected the levels of intracellular and exosomal miR-7-5p in the corresponding cells above. *P < 0.05, **P < 0.01, ***P < 0.001.

MIR-7-5p was significantly down-regulated in NSCLC cell A549, H358, H520, and SPC-A1 after Everolimus treatment (Fig. 1A), which kept more than 48 h without autophagy in NSCLC cell lines (Fig. S1A). To investigate the reduction of miR-7-5p, we analyzed the lung cancer mRNA data of TCGA, and divided the data into mTOR^low^ group and mTOR^high^ group according to the mean value of mTOR level in the samples. The gene set enrichment analysis (GSEA) on the mRNA profiles changes revealed negative associations between mTOR and gene sets were involved in extracellular exosomes (Fig. 1B). The exosomes derived from NSCLC cells in the culture medium were isolated and identified by transmission electron microscope (TEM) (Fig. 1C) and NanoSight Analysis (Fig. 1D). The size and morphology of exosomes in two groups were similar, presented as 50-100 nm round like. Exosomal markers CD63 and HSP70 in the cytoplasm was significantly weakened after Everolimus exposing (Fig. 1E). Meanwhile, HSP70, TSG101, CD63, and CD9 were significantly enriched in exosomes while intracellular exosomal markers in A549 cells were significantly decreased (Fig. 1F). But when the secretion of exosomes was inhibited by GW4869, miR-7-5p distribution was reversed (Fig. 1G).

Furthermore, we performed siTSC1/2 to active while performed Everolimus to inhibit the activity of mTORC1 [15]. And found miR-7-5p was more enriched in exosomes than in cells when Everolimus was employed. On the contrary, miR-7-5p was located in cells rather than in exosomes when siTSC1/2 was performed. Meanwhile, miR-7-5p was enriched in exosomes again when siTSC1/2-A549 cells were treated by Everolimus (Figs. 1H and S1B).

Rab27A and Rab27B are two closely related Rab small GTPases, which play key roles in exosomes secretion in many types of cells [16]. The knockdown of Rab27A and Rab27B by siRNA reduces exosome secretion of HeLa cells. Rapamycin targeting mTORC1 stimulates the release of exosome dependent on Rab27A [14]. Our results confirmed that the release of miR-7-5p-loaded exosomes was significantly decreased to almost undetectable when Rab27A or Rab27B was knocked down (Fig. S1C), accompanied by the enrichment of intracellular miR-7-5p. When Everolimus treated the cells after Rab27A or Rab27B knocking down, the exocytosis of miR-7-5p loaded exosomes was also suppressed (Fig. 1I).

### Loss of intracellular miR-7-5p induced phosphorylation of MNK/eIF4E axis, but a supplement of extra exosomal miR-7-5p could reverse it

**Fig. 2:**
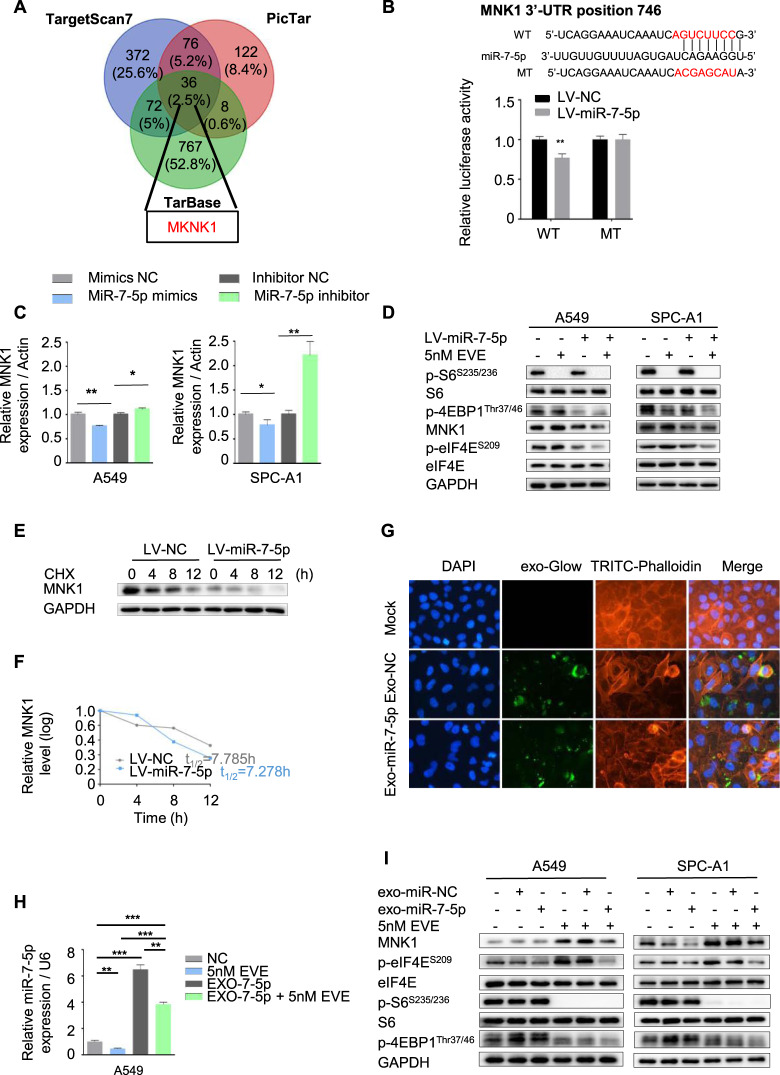
Loss of intracellular miR-7-5p induced phosphorylation of MNK/eIF4E axis, but supplement of extra exosomal miR-7-5p could reverse it. **A** The *MKNK1* was the target gene of miR-7-5p predicted by the bioinformatics analysis. **B** The binding sites of miR-7-5p in 3′-UTR region of *MKNK1*. Wild-type and mutant sequences were indicated (upper). The LV-miR-7-5p and control A549 cells were transfected with a luciferase reporter containing the 3ʹ-UTR (WT or Mut) of *MNK1*, indicating *MKNK1* was the target gene of miR-7-5p. **C** Changing the levels of miR-7-5p in A549 and SPC-A1 cells by indicated mimics or inhibitor for 24 h, the mRNA of *MKNK1* were determined by qPCR. **D** The LV-miR-7-5p and control A549 and SPC-A1 cells were treated with DMSO or 5 nM Everolimus alone or in combination for 24 h to detect the expression of MNK1 and p-eIF4E^S209^ and downstream of mTORC1, including p-S6/S6 ^S235/236^ and p-4EBP1^Thr37/46^, by Western blotting. **E** The LV-miR-7-5p A549 cells were treated with cycloheximide (20 μg/mL) and collected in the indicated times. The protein of MNK1 was detected by Western blotting. **F** The half-life of the MNK1 protein was calculated. **G** Fluorescently labeled exosomes derived from indicated A549 cells were internalized by A549 cells (exo-NC means exosomes derived from A549 infected with LV-NC; exo-miR-7-5p means exosomes derived from A549 infected with LV-miR-7-5p). Representative images were filmed after cells were fixed and stained (magnification, 400×). **H** The miR-7-5p level was detected by qPCR in A549 cells treated with Everolimus or miR-7-5p loaded exosomes alone or in combination. **I** The expression of MNK1 and p-eIF4E^S209^ and downstream of mTORC1 including p-S6/S6 ^S235/236^ and p-4EBP1^Thr37/46^, in A549 and SPC-A1 cells treated with Everolimus or miR-7-5p loaded exosomes alone or in combination were detected by Western blotting.

It had been found that feedback activation of multiple kinases during the employment of Everolimus, such as hyperphosphorylation of eIF4E at the Ser209 site, leading to drug resistance [17]. The mTOR inhibitor Rapalog induced eIF4E phosphorylation by MNKs [18]. Coincidentally, *MKNK1* was a potential targeted gene of miR-7-5p by three online analysis tools (including Pictar, TargetScan, and Tarbase) (Fig. 2A and Table S1). It could directly bind to the 3’UTR of mRNA of *MKNK1* by the luciferase reporter gene test (Fig. 2B). When miR-7-5p was elevated by mimics, the expression of MNK1 decreased. However, inhibition of miR-7-5p could up-regulate the MNK1, as shown in the Fig. 2C and Fig. S1D. A549 and SPC-A1 cell lines with lower level of miR-7-5p but higher expression of MNK1 were hired to conduct the following experiments to compare with the human bronchial epithelial (HBE) cell and other NSCLC cell lines (Fig. S1E). Lentivirus-based system (LV-miR-7-5p and LV-NC) was used to conduct stable elevated level of miR-7-5p in A549 and SPC-A1 cell lines. Higher level of miR-7-5p not only suppressed the MNK/eIF4E axis activation, but also inhibited upregulation of MNK1 and p-eIF4E^S209^ induced by Everolimus without rebound of the p-S6^S235/236^, downstream of mTOR. However, it was slightly different of p-4EBP1^Thr37/46^. In the A549 and SPC-A1 cell lines treated by Everolimus only, Everolimus could inhibit the expression of p-4EBP1^Thr37/46^ in SPC-A1 cells, but the changes in A549 cells were not obvious. And both in the LV-miR-7-5p A549 and SPC-A1 cells, p-4EBP1^Thr37/46^ was suppressed (Fig. 2D).

Simultaneously, considering higher miR-7-5p inhibits ubiquitin-mediated protein degradation [19], and MNK1 protein is degraded by ubiquitination in addition to be regulated by phosphorylation [20]. Therefore, we calculated the half-life of MNK1 protein in the LV-miR-7-5p and LV-NC A549 cells treated with protein synthesis inhibitor cycloheximide (CHX). The half-life of the MNK1 protein between the two groups was relatively close. As show in Fig. 2E and 2F, the MNK1 protein decreased by ~50% within 7.785 h in the LV-NC A549 cells, and 7.278 h in the LV-miR-7-5p A549 cells. We explored miR-7-5p-loaded exosomes derived from LV-miR-7-5p A549 cells as a candidate therapy strategy. Results showed that they were internalized by the A549 cells (Fig. 2G), and the rate of internalization is greater than the rate of secretion induced by Everolimus (Fig. 2H). They could also restrain the phosphorylation of MNK/eIF4E axis without activating the downstream of mTOR (Fig. 2I).

### The decreased miR-7-5p and elevated MNK1 were associated with a poor prognosis of NSCLC

**Fig. 3:**
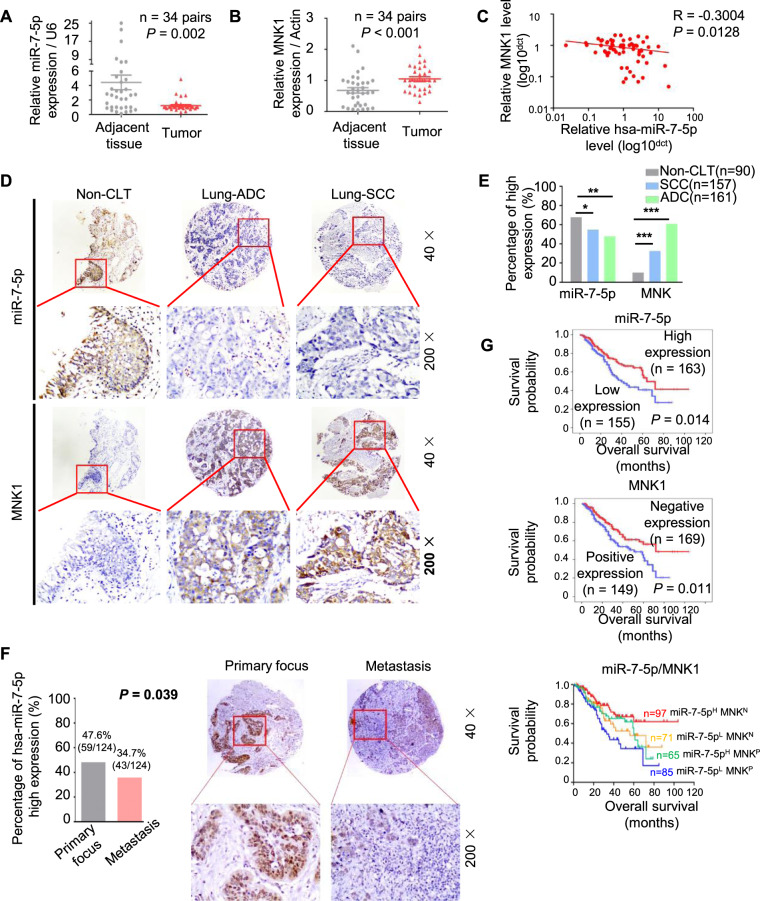
The decreased miR-7-5p and elevated MNK1 were associated with a poor prognosis of NSCLC. **A**, **B** The level of miR-7-5p and MNK1 mRNA were analyzed by RT-qPCR in 34 pairs of NSCLC tissue samples and corresponding adjacent normal lung samples. Statistical significance was calculated using the Wilcoxon test (*P* = 0.002, *P* < 0.001, respectively). **C** Correlation analysis of miR-7-5p and MNK1 mRNA levels by Spearman’s rank correlation coefficient (*R* = −0.3004, *P* = 0.0128). **D** In situ hybridization of miR-7-5p and immunohistochemistry staining for MNK1 protein in lung SCC, ADC, and non-cancerous lung tissue microarray, respectively. Both expression of miR-7-5p and MNK1 protein predominantly localized in the cytoplasm or the nucleus. Upper: Original magnification, 40×. Lower: original magnification, 200×. **E** The levels of miR-7-5p and the expression of MNK1 in lung SCC and ADC compared to non-CLT. Significant differences were observed between the groups, statistically evaluated by chi-square test. **F** *Left*. The levels of miR-7-5p in metastasis locus of NSCLC compared to primary tumors, statistically evaluated by Chi-square test (*P* = 0.039). *Right*. Representative ISH staining of miR-7-5p in primary tumors and metastasis locus of NSCLC. Upper: Original magnification, 40×. Lower: original magnification, 200×. **G** Kaplan–Meier analysis was used to plot the overall survival curves of 318 NSCLC patients with different expression of miR-7-5p and MNK1, statistical significance was assessed by log-rank test. *P < 0.05, **P < 0.01, and ***P < 0.001 compared with control.

To investigate the clinical significance of miR-7-5p and MNK1 in NSCLC tissues, we firstly confirmed the lower levels of miR-7-5p in 34 cases of NSCLC tissues compared with paired adjacent tissues by qPCR (Fig. 3A), while the mRNA of MNK1 was significantly increased in NSCLC tissues than that in paired adjacent lung tissues (Fig. 3B). There was negative correlation between the mRNA levels of miR-7-5p and MNK1 in NSCLC (Fig. 3C, *R* = −0.3004, *P* = 0.0128). We further detected miR-7-5p by in situ hybridization and MNK1 protein by immunohistochemistry in 318 cases of paraffin-embedded NSCLC tissues (including 161 cases of lung adenocarcinoma and 157 cases of lung squamous cell carcinoma) and 90 cases of adjacent lung tissues respectively. Positive expression of miR-7-5p and MNK1 was mainly located in the cytoplasm (Fig. 3D). Strongly positive miR-7-5p was observed in alveolar epithelial cells, accompanied by weak staining of MNK1 protein expression. There was low level of miR-7-5p and high expression of MNK1 protein in adenocarcinoma and squamous cell carcinoma tissues (Fig. 3E).

The low level of miR-7-5p was more prone to occur lymph node metastasis (LNM) (*P* = 0.025, Table 1) by univariate analysis. Meanwhile, the miR-7-5p were lower in metastases than that in the primary cancer (Fig. 3F). Besides, low level of miR-7-5p (*P* = 0.048) or positive expression of MNK1 (*P* = 0.007) had poor survival status (Table 1). High level of miR-7-5p combined with negative MNK1 would behave as a better survival status (*P* = 0.004) (Table 1). Overall survival (OS) for NSCLC patients with low level of miR-7-5p, positive MNK1 expression, and the combined index of miR-7-5p and MNK1 were analyzed, respectively (Fig. 3G). The NSCLC patients with low level of miR-7-5p (Fig. 3G, upper) or positive expression of MNK1(Fig. 3G, middle) tended to have poor prognosis. Optimal outcomes were observed in the patterns of combined higher miR-7-5p and negative MNK1. However, the coexistence of low level of miR-7-5p and positive expression of MNK1 indicated the worst prognosis. No statistical difference of prognosis was found in other phenotypes (Fig. 3G, lower). Compared with the primary lesion, lower miR-7-5p levels were more likely to appear in the metastatic lesions (Fig. 3H). MIR-7-5p and MNK1 were also independent prognostic factors for NSCLC regardless of LNM status, clinical stages, gender, and pathological grades by multivariate regression analysis (Table 2).

**Table 1:** Analysis of the association between expression of hsa-miR-7-5p and MNK and clinicopathological features of NSCLC (*n* = 318).

Clinicopathological features (n)	miR-7-5p			MNK			miR-7-5p/MNK^#^		
	Low (%)	High (%)	*P*-value	Po (%)	Ne (%)	*P*-value	N^−^ (%)	P^+^ (%)	*P*-value
**Age (years)**									
≤55 (*n* = 149)	70 (49.3)	72 (50.7)	0.859	68 (47.9)	74 (53.1)	0.740	38 (26.8)	104 (73.2)	0.159
> 55 (*n* = 169)	85 (48.3)	91 (51.7)		81 (46.0)	95 (54.0)		60 (34.1)	116 (65.9)	
**Gender**									
Male (*n* = 241)	116 (48.1)	125 (51.9)	0.701	108 (44.8)	133 (55.2)	0.197	79 (32.8)	162 (67.2)	0.180
Female (*n* = 77)	39 (50.6)	38 (49.4)		41 (53.2)	36 (46.8)		19 (24.7)	58 (75.3)	
**LNM status**									
NO LNM (*n* = 131)	54 (41.9)	75 (58.1)	0.042*	55 (42.6)	74 (57.4)	0.213	47 (36.4)	82 (63.6)	0.073
LNM (*n* = 187)	101 (53.4)	88 (46.6)		94 (49.7)	95 (50.3)		51 (27.0)	138 (73.0)	
**Differentiation**									
Well/Moderate (*n* = 145)	71 (49.0)	74 (51.0)	0.942	74 (51.0)	71 (49.0)	0.172	42 (29.0)	103 (71.0)	0.513
Poor (*n* = 173)	84 (48.6)	89 (51.4)		75 (43.4)	98 (56.6)		56 (32.4)	117 (67.6)	
**Clinical stages**									
StageI-II (*n* = 166)	76 (45.8)	90 (54.2)	0.270	77 (46.4)	89 (53.6)	0.861	56 (33.7)	110 (66.3)	0.239
Stage III (*n* = 152)	79 (52.0)	73 (48.0)		72 (47.4)	80 (52.6)		42 (27.6)	110 (72.4)	
**Survival status**									
Alive (*n* = 210)	94 (44.8)	116 (55.2)	0.048*	87 (41.4)	123 (58.6)	0.007**	76 (36.2)	134 (63.8)	0.004**
Dead (*n* = 108)	61 (56.5)	47 (43.5)		62 (57.4)	46 (42.6)		22 (20.4)	86 (79.6)	

^*^Chi-square test, statistically significant difference (*P < 0.05, **P < 0.01).

*ADC* adenocarcinoma, *H* High expression, *L* Low expression, *Po* Positive expression, *Ne* Negative expression, *LNM* lymph node metastasis, *NSCLC* non-small cell lung cancer, *SCC* squamous cell carcinoma, *miR-7-5p/MNK*^*#*^*, N*^*-*^ the higher miR-7-5p combined with negative expression of MNK.

P^+^, other combination of expression of these two factors.

**Table 2:** Summary of multivariate of Cox proportional regression for overall survival in 318 cases of NSCLC.

Parameter	B	SE	Wald	Sig.	Exp (B)	95.0% CI for Exp (B)	
						Lower	upper
Age	−0.153	0.199	0.593	0.441	0.858	0.580	1.268
Gender	−0.521	0.249	4.357	0.037*	0.594	0.364	0.969
Histological types	−0.040	0.215	0.034	0.853	0.961	0.630	1.465
LNM status	−0.976	0.252	15.020	0.000***	0.377	0.230	0.617
Clinical stages	−0.634	0.216	8.632	0.003***	0.530	0.347	0.810
Pathological grades	−0.486	0.202	5.801	0.016*	0.615	0.414	0.913
hsa-miR-7-5p	0.394	0.199	4.007	0.045*	1.491	1.008	2.204
MNK	−0.464	0.206	5.095	0.024*	0.629	0.420	0.941

*CI* confidence interval, *LNM* lymph node metastasis, *NSCLC* non-small cell lung cancer.

Note: multivariate analysis of Cox regression, *P < 0.05, ***P < 0.001.

Moreover, Spearman’s rank correlation analysis investigated the association between miR-7-5p and MNK1 in 318 cases of NSCLC (Table 3). Among 318 cases of NSCLC cases, 71 patients had low level of miR-7-5p and negative expression of MNK1 commonly. Both positive expression of MNK1 and high level of miR-7-5p in 65 patients. However, 98 cases had negative expression of MNK1 but high level of miR-7-5p, 84 cases had positive expression of MNK1 but low level of miR-7-5p. There was statistically negative correlation between miR-7-5p and MNK1 (*r* = −0.143, *P* = 0.011).

**Table 3:** The pairwise association between expression of miR-7-5p and MNK in 318 cases of NSCLC.

MNK			
	Positive (%)	Negative (%)	*P*-value
**miR-7-5p**			
High (%)	65 (39.9)	98 (60.1)	0.011*
Low (%)	84 (54.2)	71 (45.8)	(*r* = −0.143)

^*^Spearman’s rank correlation test, statistically significant difference (*P < 0.05)

### Exosomal miR-7-5p enhanced the anticancer effect of Everolimus in vitro

**Fig. 4:**
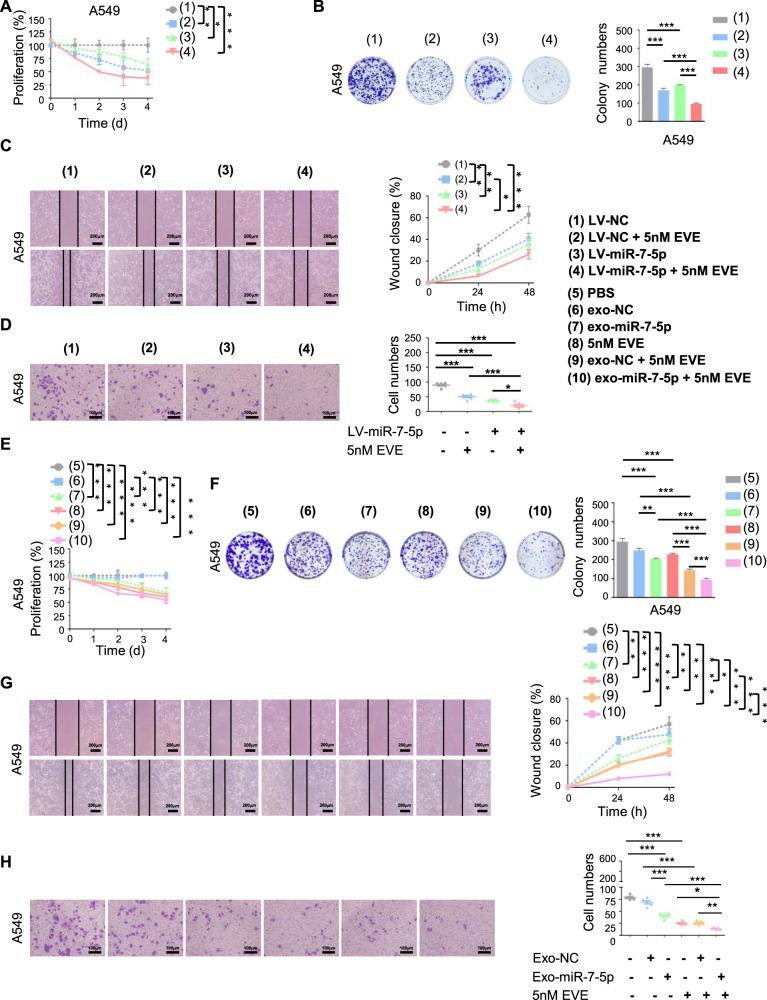
Exosomal miR-7-5p enhanced the anticancer effect of Everolimus in vitro. Indicated A549 cells were treated with or without Everolimus at the settled time. The cell proliferative ability was determined by **A** CCK8 assay and **B** cell clone-formation assay. The cell migration and invasive ability was determined by **C** cell scratch test and **D** transwell matrigel assay. A549 cells were treated with indicated exosomes or Everolimus for settled times. The cell proliferative ability was determined by **E** CCK8 assay and **F** cell clone-formation assay. The cell migrating and invasive ability was determined by **G** cell scratch test and **H** transwell matrigel assay. Data are shown as mean ± SD. *P < 0.05, **P < 0.01, and ***P < 0.001 compared with control.

Our results also indicated that Everolimus alone or in combination with high level of miR-7-5p could inhibit proliferation (Figs. 4A and S2A), clone formation (Figs. 4B and S2B), and 2D&3D migration capabilities (Figs. 4C, D and S2C–S2D, respectively) of NSCLC cells. The inhibitory effect was the most significant in the stable miR-7-5p cells with Everolimus treatment.

The exosomes have been proposed as a potential cell-based alternative therapy [21]. We used exosomes derived from A549 cells directly to improve miR-7-5p in NSCLC cells with Everolimus treatment because the direct sorting and packaging of nucleic acids into exosomes may not provide the functionally active contents into recipient cells effectively [22]. Like exogenous miR-7-5p, exosomal miR-7-5p could inhibit the proliferation (Figs. 4E and S2E), clone formation (Figs. 4F and S2F), and 2D&3D migration capabilities (Figs. 4G, H and S2G–S2H, respectively) of NSCLC cells. In addition, the combination of miR-7-5p-loaded exosomes with Everolimus showed the obvious anticancer effect.

### Exosomal miR-7-5p enhanced the anticancer therapeutic efficacy of Everolimus in vivo

**Fig. 5:**
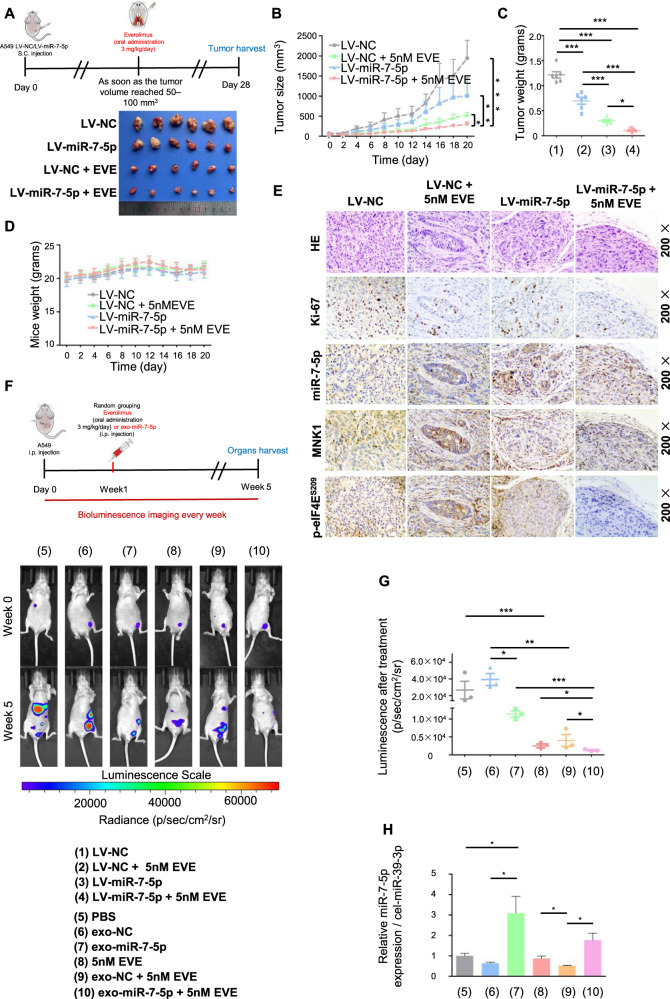
Exosomal miR-7-5p enhanced the anticancer therapeutic efficacy of Everolimus in vivo. Five-week-old male nude mice were randomly divided into two groups to establish LV-NC and LV-miR-7-5p A549 cells subcutaneous xenograft nude mice models. As soon as the tumor volume reached 50-100mm^3^, they would be divided into four groups named as 1) The LV-NC group, 2) The LV-miR-7-5p group, 3) The LV-NC combined Everolimus group and 4) The LV-miR-7-5p combined Everolimus group. **A** Upper: Schematic illustration of Everolimus oral administration for the subcutaneous xenograft nude mice models. Lower: Comparison of tumor engraftment size and weight in nude mice subcutaneously injected into the flanks with indicated A549 cells and indicated treatment. **B** The mice xenograft tumor growth curves of the four groups, **C** the tumor weight and **D** the body weight. **E** Immunohistochemical/In situ hybridization staining for the proliferation marker Ki67, MNK1, p-eIF4E^S209^ and miR-7-5p in the tumor tissues. The A549 cells labeled with luciferase gene were injected into the abdominal cavity of nude mice, and the baseline level of tumor cells was monitored by live imaging technology immediately after injection. One week later, they would be randomly divided into 6 groups for receiving PBS, exo-NC, exo-miR-7-5p, Everolimus, exo-NC combined with Everolimus and exo-miR-7-5p combined with Everolimus treatment, respectively. **F** Upper: Schematic illustration of abdominal tumor xenograft nude mice models. Lower: Luminescence signals of intraperitoneal A549-luciferase cells with different treatment groups at the indicated week. Results are shown as the mean ± SD. **G** Analysis of intraperitoneal A549-luciferase cells accepted different treatment groups at the indicated week. **H** The levels of exo-miR-7-5p from the plasma of nude mice receiving treatments above were measured by qPCR. * *P* < 0.05, ** *P* < 0.01, ***P < 0.001.

Nude mouse subcutaneous tumor models were established with LV-miR-7-5p and LV-NC A549 cells, respectively (Fig. 5A). And we divided them into 4 groups to accept further Everolimus exposure or not. The tumor volume (Fig. 5B), the growth rate (Fig. S3A), and the tumor weight (Fig. 5C) of the LV-miR-7-5p group were significantly slower and smaller than that of the control group. Although Everolimus obviously inhibited tumor growth in 10 days before, the growth rate tended to accelerate in the later period (Fig. S3B), resulting in the bigger tumors volume of the LV-NC group than that of the LV-miR-7-5p combined Everolimus. The cells proliferation and tumor volume always kept at a lowest level when LV-miR-7-5p was performed with Everolimus (Fig. 5A). There were no significant changes of the body weight of nude mice in the above treatment (Fig. 5D). Next, we detected the expression of Ki-67, MNK1, and p-eIF4E^S209^ and the level of miR-7-5p in the Xenograft tumors. Results indicated that though Everolimus could suppress the Ki-67 index, representing proliferation ability of cells, in combined with LV-NC or LV-miR-7-5p group, the overexpression of MNK1 and p-eIF4E^S209^ was only observed in the LV-NC combined Everolimus group (Fig. 5E and Fig. S3C).

Because of safer biological behaviors, exosomes have the value of translational medicine for the selective delivery of anticancer therapies [22]. We further established the abdominal metastasis models accepting the indicated treatments. Tumor involvement was observed in all groups (Fig. 5F and Fig. S3D). Through quantifying the fluorescence intensity involved in abdominal metastases, we found both exosomal miR-7-5p and Everolimus could inhibit the growth of abdominal metastases. Notably, the exo-miR-7-5p combined with Everolimus had the weakest fluorescence signal (Fig. 5G), and high level of miR-7-5p in the blood (Fig. 5H).

### Combination of miR-7-5p with Everolimus induced apoptosis to exhibit a synergistic anticancer therapeutic efficacy through dual abrogation of MNK/eIF4E and mTOR in NSCLC

**Fig. 6:**
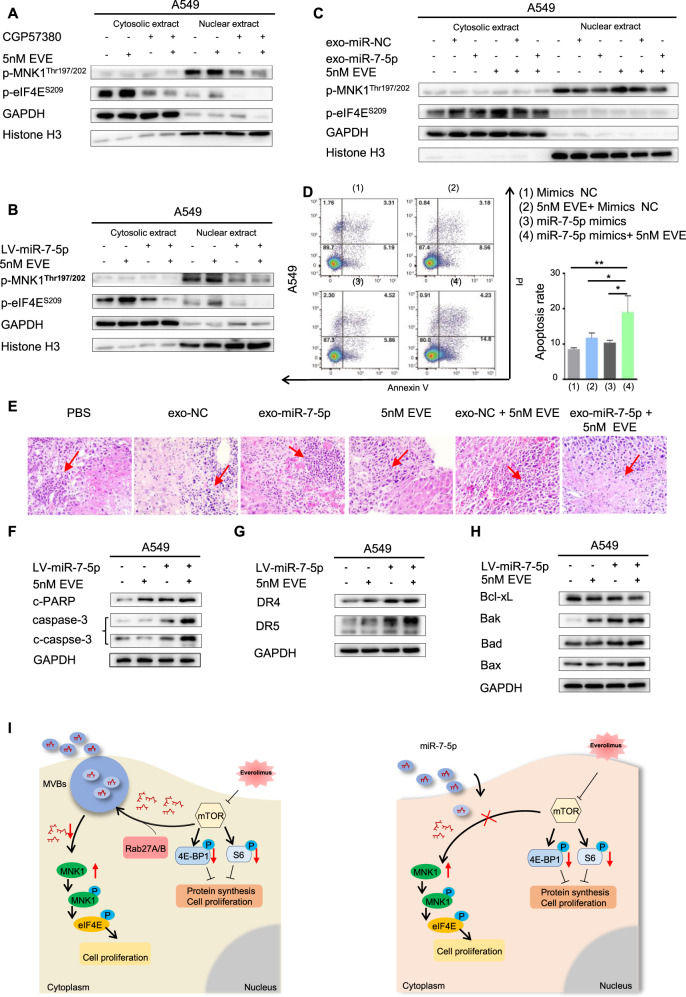
Combination of miR-7-5p with Everolimus induced apoptosis to exhibit a synergistic anticancer therapeutic efficacy via dual abrogation of MNK/eIF4E and mTOR in NSCLC. Analysis of proteins of p-MNK1 ^Thr197/202^ and p-eIF4E^S209^ respectively extracted from cytosolic or nuclear in the indicated A549 cells was detected using Western blotting. GAPDH was used as a loading cytoplasmic control. And Histone H3 was used as a loading nuclear control. **A** A549 cells were treated with MNK inhibitor CGP57380 or mTORC1 inhibitor Everolimus alone or in combination. **B** The LV-miR-7-5p or LV-NC A549 cells were treated with or without Everolimus. **C** Treatment of A549 cells with Everolimus or miR-7-5p loaded exosomes alone or in combination. **D** Cells were transfected with indicated mimics with or without Everolimus for 48 h. Apoptotic cells were analyzed by flow cytometry using Annexin V/ PI staining. Columns, means of three replicate determinations; each bar represents mean ± SD. *P < 0.05, ** *P* < 0.01. **E** Representative microscopic images of liver metastatic lesions among all groups in previous abdominal metastasis models. The arrow showed the metastasis. **F**–**H** The stably LV-miR-7-5p of A549 cells were treated with Everolimus or DMSO for 48 h. Cell lysates were harvested for western blotting analysis to detect the indicated apoptotic proteins, and GAPDH was used as a loading control. **I** Schematic representation of a model for the major molecular mechanisms of “Exosome-mediated miR-7-5p delivery enhances the anticancer effect of Everolimus via blocking MNK/eIF4E axis” in NSCLC.

The function of eIF4E is determined by its subcellular locations. In the initial phase of translation, most mRNAs are controlled by intracytoplasmic eIF4E [23]. We further used MNK1 inhibitor CGP57380 to explore the mechanism of combination of targeting of the MNK/eIF4E axis and the mTOR pathway in NSCLC. We found p-MNK1^Thr197/202^ was mainly distributed in the nucleus and was inhibited. CGP57380 mainly suppressed the phosphorylation of intracytoplasmic eIF4E (Fig. 6A and Fig. S4A). Because of poor specificity and relatively weak affinity, CGP57380 in clinical treatment was limited [24]. Fortunately, both endogenous and exosomal miR-7-5p could restore the intracellular miR-7-5p and reverse the feedback activation of MNK/eIF4E axis more specific and safer. When stable LV-miR-7-5p A549 and SPC-A1 cells were treated by Everolimus (Fig. 6B and Fig. S4B) or combination of Everolimus with the miR-7-5p-loaded exosomes derived from A549 cells (Fig. 6C and Fig. S4C), the activation of the MNK/eIF4E axis was eliminated.

The apoptosis rate of miR-7-5p mimics or Everolimus treatment alone was less than that of the combined group (Fig. 6D and Fig. S4D) using flow cytometry analysis. Representative microscopic images of liver metastatic lesions among all groups in previous abdominal metastasis models showed obvious necrosis in exo-miR-7-5p combined with Everolimus group (Fig. 6E). Caspase-3, cleaved caspase-3, DR4, DR5, pro-apoptotic proteins Bak, Bad and Bax were significantly up-regulated but anti-apoptotic protein Bcl-xL was down-regulated (Fig. 6F–H and Fig. S4E–G).

## Discussion

Everolimus is a derivative of rapamycin, which inhibits the activity of mTORC1 as a potential therapeutic drug in cancers. The single drug activity in the cancers is limited [25]. Targeted mTOR can feedback-activate a variety of cell survival signaling pathways including PI3K/Akt, MEK/ERK and MNK/eIF4E to attenuate rapamycin treatment [17]. In addition, long-term effects of rapamycin lead to changes of intracellular miRNA expression profile such as increased oncogenic miRNAs and decreased tumor suppressor miRNAs. mTOR inhibitors promotes the excretion of miRNA into the cellular microenvironment to neutralize its antitumor activity [26]. We confirmed that Everolimus decreased miR-7-5p in NSCLC, which was consistent with published research [9]. The bioinformatics and luciferase reporter assay validated that miR-7-5p could target the mRNA of *MKNK1*. mTOR inhibitors induce the eIF4E phosphorylation in a MNK-dependent manner [27]. Our results firstly confirmed that the up-regulation of miR-7-5p combined with Everolimus could indeed inhibit the phosphorylation of the MNK/eIF4E axis induced by Everolimus without feedback activating of p-S6^S235/236^. We also noticed that the expression of p-4EBP1^Thr37/46^ in the A549 and SPC-A1 cell lines treated by Everolimus only, it could be inhibited in SPC-A1 cells, but changed slightly in A549 cells. This is mainly due to the heterogeneity of two cell lines, and the difference in processing conditions. Our previous study found that the expression of p-4EBP1^Thr37/46^ could be inhibited in A549 cells after 24 and 48 h of Everolimus treatment [28]. In order to ensure the consistency of other experimental conditions in this study, we only processed 12 h of Everolimus. As for the inhibition of miR-7-5p on the p-4EBP1^Thr37/46^, no clear mechanism has been found yet, and we may need to study further in the future. If the upregulation of miR-7-5p inhibits ubiquitinated degradation of MNK1, it would offset the tumor suppressor effect of miR-7-5p [19]. Hence, we further verified that the targeted inhibitory effect of miR-7-5p on MNK protein was independent on the proteasome mediated degradation of MNK1. For the first time, our studies indicated that Everolimus targeted mTORC1 to induce the loss of intracellular miR-7-5p for activating MNK/eIF4E axis, thus impairing the effect of Everolimus in NSCLC.

In NSCLC, the role of miR-7 was contradictory [29]. We found decreased miR-7-5p in cell lines and tissues of NSCLC, which was related to the poor prognosis of patients. Elevated miR-7-5p inhibited the proliferation, growth and migration of NSCLC in vivo and in vitro. The contradictory functions of miR-7-5p might be explained by the balance of the positive or negative feedback loops in different cancer types or stages.

The transfer of miRNAs by exosomes is considered a novel and important mechanism of genetic exchange between cells [30]. Hence, according to analysis of TCGA database, we speculated that Everolimus could stimulate NSCLC cells to excrete miR-7-5p-loaded exosomes, thereby reducing intracellular miR-7-5p to weaken the anti-tumor activity of Everolimus. The mTORC1 also regulated the release of exosomes depending on two crucial exosomes regulatory proteins, Rab27A and Rab27B [31]. This study proved that Everolimus induced exosomes derived from NSCLC cells enrich miR-7-5p. There might be a mechanism involved in the sorting process of miR-7-5p into exosomes derived from Everolimus treated NSCLC cells. The motif of “GUUG” was associated with indicated miRNAs into exosomes in SW620 cell [32]. The base sequence of miR-7-5p (UGGAAGACUAGUGAUUUUUGUUGU) contains the “GUUG” motif, which might be a candidate reason for miR-7-5p sorting process needed further verification.

The exosomes could be used as carriers, which had the value of low immunogenicity, low toxicity, and high stability through being modified to improve loading and targeting efficiency [33]. Restoring normal miRNA levels are regarded as novel therapeutic intervention in the Everolimus treatment [34]. In this study, the efflux exosomes derived from miR-7-5p elevated NSCLC cells had higher content of miR-7-5p, which could be internalized by A549 cells. We further confirmed in vitro and in vivo that miR-7-5p-loaded exosomes derived from A549 cells were provided with important potential for the tumor treatment. The results were consistent with the previous preclinical studies [35]. Although there was no definitive result at present, some clinical trials were ongoing to evaluate the efficacy of miRNA and exosomes as therapeutic agents [36].

The confounding reality for anti-cancer drug development is the bewildering adaptive aptitude of tumor cells [37]. Activating redundant and alternative signaling pathways is one of the recent insights into adaptive anti-cancer drug resistance mechanisms. The majority of the resistance mechanisms associated with mTOR inhibitor belongs to this kind of adaptive resistance phenomenon [38]. In this study, we discovered that although Everolimus obviously inhibited tumor growth in 10 days before, the growth rate tended to accelerate in the later period. Further immunohistochemistry test identified activation of MNK/eIF4E existed in Everolimus therapy. The MNK deletion reduces mTORC1 signaling, and MNK activation contributes to rapamycin resistance in cancer cells [39]. However, little progress was found in clinical treatment due to the MNK inhibitory effect of CGP57380 [40]. It is urgent to explore new effective methods to target the MNK/eIF4E axis. The miRNA analogs or anti-miRNAs is serviced as innovative anti-cancer strategies. Here, we found that miR-7-5p loaded exosomes could combine with Everolimus to induce apoptosis in the mitochondrial pathway and the death receptor pathway through dual targeting the MNK/eIF4E axis and mTOR pathway. The strategy of miR-7-5p loaded exosomes combined with Everolimus was more effective than our previous combination of CGP57380 with Everolimus in NSCLC, which only promoted apoptosis through the mitochondrial pathway [10]. Enforced levels of exogenous miR-7 in TRAIL-overexpressed MSCs sensitize the treatment of TRAIL to increase apoptosis in the death receptor pathway through targeting XIAP in glioma cells [41]. We speculated that the up-regulation of miR-7-5p was associated with death receptor pathway-mediated apoptosis, which provided important support for the clinical application of tumor suppressor miR-7-5p.

In summary, Everolimus stimulates the release of miR-7-5p loaded exosomes from NSCLC cells in Rab27A and Rab27B-dependent manners. Everolimus decrease intracellular miR-7-5p attenuating the inhibition of MNK1 and promoting MNK-dependent eIF4E phosphorylation to blunt its effectiveness in NSCLC. Either endogenous miR-7-5p or exo-miR-7-5p combined with Everolimus synergistically enhance the anticancer efficacy through targeting MNK/eIF4E axis and mTOR pathway (Fig. 6I). In addition, both low level of miR-7-5p and positive expression of MNK1 act as independent poor prognostic biomarkers for NSCLC. Therefore, restoration of miR-7-5p carried by exosome may be considered as a promising novel combined therapeutic strategy with Everolimus for NSCLC.

## Materials and methods

We describe antibodies, reagents and cells in details in the Supplemental Material and Methods. All in vitro experiments were repeated three times.

### Clinical data and tissue microarrays (TMA)

In this study, a total of 318 cases of NSCLC and 91 cases of non-cancerous lung tissue (Non-CLT) were chosen randomly from The Second Xiangya Hospital of Central South University (Changsha, China). Complete clinical records and follow-up data were available for all patients. Besides, 40 pairs of primary NSCLC tissues and adjacent non-tumor lung tissues were collected from 40 cases of NSCLC. Protocols were approved by The Second Xiangya Hospital of Central South University Ethics Review Board (Scientific and Research Ethics Committee, No. K021/2021). All patients involved in our study had written informed consent. The staging classification of this research was carried out based on the criteria of the 8th edition of the AJCC/UICC TNM staging system of lung cancer (2017). The confirmed histological diagnosis of NSCLC according to the World Health Organization histological classification of lung cancer. No patients had been previously treated with chemotherapy and radiotherapy at the time of original operation. Complete clinical record and follow-up data were available for all patients. We used the TMA technology designed and constructed high-throughput NSCLC TMAs according to rules previously described [42].

### Quantitative PCR analysis

We extracted total RNA using TRIzol reagent (Invitrogen). Two μg of total RNA was performed to synthesize cDNA using by Mir-X miRNA First-Strand Synthesis Kit (Takara) or RevertAid First-Strand cDNA Synthesis Kit (Thermo) according to the manufacturer’s protocol. The RNA level was measured by qPCR with a TB Green premix Ex Taq II kit (Takara) or 2 × SYBR Green qPCR Master Mix (Bimake) on CFX96 Real-Time PCR Detection System (Bio-Rad). Relative expression was determined with a U6 control or β-actin through the 2^-ΔΔ^ Ct method.

Before isolation of RNA from exosomes, 1 × 10^8^ copies/μL of C. elegans cel-miR-39-3p mimics (Qiagen) was added to samples as a spike-in control.

### Western blotting

Whole-cell protein lysates preparation and Western blotting analysis were performed as described previously [43]. Cytoplasmic and nuclear extracts were obtained by using the CEB-NEB kit (Applygen, China) according to the manufacturer’s protocol.

### Exosome isolation and identification

Before cell culture, FBS was depleted of exosomes by ultracentrifugation at 1 × 10^5^ g at 4 °C for 12 h, then the supernatant was harvested and filtered with a 0.22 um filter (Millipore). After 24 h, the cell culture medium with exosome-free FBS was collected and removed floating cells and cellular debris from the supernatant by centrifuging at 3000 g for 30 min. The supernatant was then passed through a 0.22 μm filter. The resultant supernatant was forced through the membrane by centrifugal filtration at 3000 *g* for 1 h with the ultrafiltration device (UFC900396, Millipore). Finally, the total exosome isolation reagent (from cell culture media, Invitrogen) was added to the concentrated solution at a ratio of 1:2. Subsequent exosome extraction was conducted according to the manufacturer’s protocol. The exosomes were used for the following experiments immediately or stored at -80 °C. Exosomal protein was measured by the BCA™ Protein Assay Kit (Thermo). The concentration of exosomal proteins was quantified using a BCA protein assay kit (Keygen Biotech, KGP902, China). HSP70, TSG101, CD63 and CD9 expression were measured using western blot analysis, and GAPDH as an internal reference.

Isolated exosomes were fixed in 1% glutaraldehyde for 10 min, washed with deionized water. 10μL of exosome suspension was placed on formvar carbon-coated 300-mesh copper electron microscopy grids, incubated for 5 min at room temperature and air-dried for 5 min. Images were obtained by TEM [44]. In addition, exosomes were examined via injecting into the Zetasizer Nano ZS90 instrument. Phosphate buffered saline was used as controls. Data was analyzed by Zetasizer software.

### Exosome labeling

Exosomes were resuspended in 500 μL PBS mixed with 1 μL of the 500 × labeling dye (ExoGlow protein EV Labeling Kit, SBI) and incubated at 37 °C for 30 min. Then 167 μL ExoQuick-TC (SBI) was added to stop the reaction and incubated at 4 °C overnight. Labeled exosomes were isolated from the supernatant by centrifugation of 10000 rpm for 30 min and resuspended in PBS.

Fluorescently labeled exosomes were added to A549 cells which were at 80% confluence and incubated overnight. Cells were fixed with 4% paraformaldehyde for 30 min at room temperature and then permeabilized with 0.25% Triton-X for 30 min. Next, the cells were stained with TRITC phalloidin for 30 min and 4’, 6-diamidino-2-phenylindole (DAPI) for 5 min sequentially. Finally, cells were washed twice with PBS to remove excess DAPI. A549 cells that up took the labeled exosomes were observed under a fluorescence microscope.

### Cell transfection

The synthetic miRNA mimics, inhibitors, negative control (NC), siTSC1, siTSC2, siRab27A or siRab27B (GenePharma, Shanghai, China) were mixed with lipofectamine 3000 (Invitrogen) and then added to the cell culture medium according to the manufacturer’s instructions. After 24 h or 48 h of transfection, total RNA and protein were extracted from cells, respectively.

### Lentivirus transduction

The LV-miR-7-5p and the control virus LV-NC were purchased from GenePharma (Shanghai, China). Cells were infected with lentivirus for 2 days and then added to puromycin (2.5 mg/mL) for 2 weeks to generate stably transduced cell lines.

### Dual-luciferase reporter assay

The full length of MNK1 3’-UTR were amplified and subcloned into the pmirGLO dual-luciferase miRNA target expression vector. The wild type and mutant sites in MNK1 3’-UTR or eIF4E 3’-UTR were produced by GeneCreate Biotech (Wuhan, China). Luciferase activity assays were performed with the Dual-Luciferase Reporter Assay System (Promega, Madison, WI).

### Xenograft mouse tumor models

For the subcutaneous tumor model, the LV-miR-7-5p and LV-NC A549 cells (1 × 10^7^ cells) in 200 μL serum-free medium were injected subcutaneously into female BALB/c nude mice, respectively. As soon as the tumor volume reached 50-100 mm^3^, mice would be divided into four groups (*n* = 6/group) named as the LV-NC group, the LV-miR-7-5p group, the LV-NC combined Everolimus (3 mg/kg/day, oral gavage) group and the LV-miR-7-5p combined Everolimus group, randomly. The four groups received indicated treatments. The size of the tumors and the weight of the mice were monitored every 2 days. After 28 days, the mice were euthanized and the tumors were obtained. Tumor volume was calculated with the formula: V = (length × width2) /2. Finally, all tumors were excised, weighed, and fixed in 10% neutral buffered formalin for 24 h, and the paraffin sections were routinely stained with hematoxylin/eosin.

As for the intraperitoneal tumor model, single-cell suspensions of luciferase tagged A549-cells (1 × 10^7^ cells) were injected into the abdominal of female BALB/c nude mice. And the baseline level of tumor cells was monitored by live imaging technology immediately after injection. And then, the survival and mental state of the nude mice were monitored every two days. After one week, we confirmed the survival of tumor tissue, randomly divided them into 6 groups (*n* = 3/group), and received PBS, exo-NC, exo-miR-7-5p, Everolimus, exo-NC combined with Everolimus and exo-miR-7-5p combined with Everolimus treatment, respectively. The development of tumor burden was monitored by bioluminescence imaging (BLI) of anesthetized mice that were intraperitoneally injected with 75 mg/kg D-luciferin (Thermo).

The implementation of the experiment was completed under double-blind conditions. Protocols were approved by The Second Xiangya Hospital of Central South University Ethics Review Board (Scientific and Research Ethics Committee, No. K021/2021) and with ethical regulations.

### Colony formation assay, CCK-8 assay, wound healing and transwell migration assays

These procedures were performed as previously described [9].

### Flow cytometry

For cell apoptosis detection, cells were plated into 6-well plates and transfected with miRNA mimics for 24 h and treated with/without 25 nM Everolimus after an additional 48 h. The number of apoptotic cells were detected using a FITC-Annexin V apoptosis detection kit (BD Pharmingen) and a flow cytometer following the manufacturer’s instructions.

### Immunohistochemistry (IHC) and in-situ hybridization (ISH)

The immunohistochemical staining for Ki-67, p-eIF4E^S209^ and MNK1 and the In-situ hybridization assay of miR-7-5p was performed as previously described [9, 45]. And the expression levels of Ki-67, miR-7-5p, p-eIF4E^S209^ and MNK1 in tissues of xenograft mouse tumor models were analyzed by the Image J Software. The statistics of the results are done double-blind.

### Statistical analysis

Statistical analyses were determined by chi-square test, Spearman correlation test, multivariate Cox regression analysis, Pearson correlation, Wilcoxon rank-sum test, or student’s t-test as appropriate using SPSS 19.0 and GraphPad Prism 5.0. Error bars indicated the standard deviation in all the figures. *P < 0.05, **P < 0.01, ***P < 0.001. And *P* value <0.05 was considered significant.

## Supplementary information

Supporting Information

Figure S1.

Figure S2.

Figure S3.

Figure S4.

Table S1.

Table S2.

Table S3.

Table S4.

Table S5.

aj-checklist

## Data Availability

All data generated or analyzed during this study are included in the main text and the supplementary information files.

## References

[1] Tan AC (2020). Targeting the PI3K/Akt/mTOR pathway in non‐small cell lung cancer (NSCLC). Thorac Cancer.

[2] Guertin DA, Sabatini DM (2007). Defining the role of mTOR in cancer. Cancer Cell.

[3] Dong S, Zhang XC, Cheng H, Zhu JQ, Chen ZH, Zhang YF (2012). Everolimus synergizes with gefitinib in non-small-cell lung cancer cell lines resistant to epidermal growth factor receptor tyrosine kinase inhibitors. Cancer Chemother Pharm.

[4] Fang W, Huang Y, Gu W, Gan J, Wang W, Zhang S (2020). PI3K-AKT-mTOR pathway alterations in advanced NSCLC patients after progression on EGFR-TKI and clinical response to EGFR-TKI plus everolimus combination therapy. Transl Lung Cancer Res.

[5] Justin S, Rutz J, Maxeiner S, Chun FK, Juengel E, Blaheta RA (2020). Bladder cancer metastasis induced by chronic Everolimus application can be counteracted by sulforaphane in vitro. Int J Mol Sci.

[6] Katsha A, Wang L, Arras J, Omar OM, Ecsedy J, Belkhiri A (2017). Activation of EIF4E by aurora kinase A depicts a novel druggable axis in Everolimus-resistant cancer cells. Clin Cancer Res.

[7] Yoshizawa A, Fukuoka J, Shimizu S, Shilo K, Franks TJ, Hewitt SM (2010). Overexpression of phospho-eIF4E is associated with survival through AKT pathway in non-small cell lung cancer. Clin Cancer Res.

[8] Totary-Jain H, Sanoudou D, Ben-Dov IZ, Dautriche CN, Guarnieri P, Marx SO (2013). Reprogramming of the microRNA transcriptome mediates resistance to rapamycin. J Biol Chem.

[9] Liu S, Zang H, Zheng H, Wang W, Wen Q, Zhan Y (2020). miR-4634 augments the anti-tumor effects of RAD001 and associates well with clinical prognosis of non-small cell lung cancer. Sci Rep..

[10] Korać P, Antica M, Matulić M (2021). MiR-7 in cancer development. Biomedicines.

[11] Webster RJ, Giles KM, Price KJ, Zhang PM, Mattick JS, Leedman PJ (2009). Regulation of epidermal growth factor receptor signaling in human cancer cells by microRNA-7. J Biol Chem.

[12] Wang Y, Liu J, Liu C, Naji A, Stoffers DA (2013). MicroRNA-7 regulates the mTOR pathway and proliferation in adult pancreatic β-cells. Diabetes.

[13] Wan M, Ning B, Spiegel S, Lyon CJ, Hu TY (2020). Tumor-derived exosomes (TDEs): how to avoid the sting in the tail. Med Res Rev.

[14] Zou W, Lai M, Zhang Y, Zheng L, Xing Z, Li T (2018). Exosome release is regulated by mTORC1. Adv Sci (Weinh).

[15] Liu W, Yi Y, Zhang C, Zhou B, Liao L, Liu W (2021). The expression of TRIM6 activates the mTORC1 pathway by regulating the ubiquitination of TSC1-TSC2 to promote renal fibrosis. Front Cell Dev Biol.

[16] Alexander M, Ramstead AG, Bauer KM, Lee SH, Runtsch MC, Wallace J (2017). Rab27-dependent exosome production inhibits chronic inflammation and enables acute responses to inflammatory stimuli. J Immunol.

[17] Sun SY (2021). mTOR-targeted cancer therapy: great target but disappointing clinical outcomes, why?. Front Med.

[18] Fan C, Zhao C, Zhang F, Kesarwani M, Tu Z, Cai X (2021). Adaptive responses to mTOR gene targeting in hematopoietic stem cells reveal a proliferative mechanism evasive to mTOR inhibition. Proc Natl Acad Sci USA.

[19] Sabater-Arcis M, Bargiela A, Furling D, Artero R (2020). miR-7 restores phenotypes in myotonic dystrophy muscle cells by repressing hyperactivated autophagy. Mol Ther Nucleic Acids.

[20] Donovan KA, Ferguson FM, Bushman JW, Eleuteri NA, Bhunia D, Ryu S (2020). Mapping the degradable kinome provides a resource for expedited degrader development. Cell.

[21] Kalluri R, LeBleu VS (2020). The biology, function, and biomedical applications of exosomes. Science.

[22] Wei H, Chen Q, Lin L, Sha C, Li T, Liu Y (2021). Regulation of exosome production and cargo sorting. Int J Biol Sci.

[23] Witzig TE, Reeder C, Han JJ, LaPlant B, Stenson M, Tun HW (2015). The mTORC1 inhibitor everolimus has antitumor activity in vitro and produces tumor responses in patients with relapsed T-cell lymphoma. Blood.

[24] Dreas A, Mikulski M, Milik M, Fabritius CH, Brzózka K, Rzymski T (2017). Mitogen-activated protein kinase (MAPK) interacting kinases 1 and 2 (MNK1 and MNK2) as targets for cancer therapy: recent progress in the development of MNK inhibitors. Curr Med Chem.

[25] Lu X, Paliogiannis P, Calvisi DF, Chen X (2021). Role of the mammalian target of rapamycin pathway in Liver cancer: from molecular genetics to targeted therapies. Hepatology.

[26] Nogueira I, Dias F, Morais M, Teixeira AL, Medeiros R (2019). Everolimus resistance in clear cell renal cell carcinoma: miRNA-101 and HIF-2α as molecular triggers?. Future Oncol.

[27] Wang X, Yue P, Chan CB, Ye K, Ueda T, Watanabe-Fukunaga R (2007). Inhibition of mammalian target of rapamycin induces phosphatidylinositol 3-kinase-dependent and Mnk-mediated eukaryotic translation initiation factor 4E phosphorylation. Mol Cell Biol.

[28] Wen Q, Wang W, Luo J, Chu S, Chen L, Xu L (2016). CGP57380 enhances efficacy of RAD001 in non-small cell lung cancer through abrogating mTOR inhibition-induced phosphorylation of eIF4E and activating mitochondrial apoptotic pathway. Oncotarget.

[29] Chou YT, Lin HH, Lien YC, Wang YH, Hong CF, Kao YR (2010). EGFR promotes lung tumorigenesis by activating miR-7 through a Ras/ERK/Myc pathway that targets the Ets2 transcriptional repressor ERF. Cancer Res.

[30] Cheng W, Wang K, Zhao Z, Mao Q, Wang G, Li Q (2020). Exosomes-mediated transfer of miR-125a/b in cell-to-cell communication: a novel mechanism of genetic exchange in the intestinal microenvironment. Theranostics.

[31] Ostrowski M, Carmo NB, Krumeich S, Fanget I, Raposo G, Savina A, et al. Rab27a and Rab27b control different steps of the exosome secretion pathway. Nat Cell Biol. 2010;12:19–30. 10.1038/ncb2000 19966785

[32] Gao T, Shu J, Cui J (2018). A systematic approach to RNA-associated motif discovery. BMC Genomics.

[33] Pi YN, Xia BR, Jin MZ, Jin WL, Lou G (2021). Exosomes: powerful weapon for cancer nano-immunoengineering. Biochem Pharmacol.

[34] Zhang J, Li S, Li L, Li M, Guo C, Yao J (2015). Exosome and exosomal microRNA: trafficking, sorting, and function. Genomics Proteom Bioinformatics.

[35] Nie H, Xie X, Zhang D, Zhou Y, Li B, Li F (2020). Use of lung-specific exosomes for miRNA-126 delivery in non-small cell lung cancer. Nanoscale.

[36] H Rashed M, Bayraktar E, K Helal G, Abd-Ellah MF, Amero P, Chavez-Reyes A (2017). Exosomes: from garbage bins to promising therapeutic targets. Int J Mol Sci.

[37] Junttila MR, de Sauvage FJ (2013). Influence of tumour micro-environment heterogeneity on therapeutic response. Nature.

[38] Ferreira BI, Lie MK, Engelsen AST, Machado S, Link W, Lorens JB (2016). Adaptive mechanisms of resistance to anti-neoplastic agents. Medchemcomm.

[39] Yang X, Zhong W, Cao R (2020). Phosphorylation of the mRNA cap-binding protein eIF4E and cancer. Cell Signal.

[40] Abdelaziz AM, Yu M, Wang S (2021). Mnk inhibitors: a patent review. Pharm Pat Anal.

[41] Zhang X, Zhang X, Hu S, Zheng M, Zhang J, Zhao J (2017). Identification of miRNA-7 by genome-wide analysis as a critical sensitizer for TRAIL-induced apoptosis in glioblastoma cells. Nucleic Acids Res.

[42] Chu S, Wen Q, Qing Z, Luo J, Wang W, Chen L (2017). High expression of heat shock protein 10 correlates negatively with estrogen/progesterone receptor status and predicts poor prognosis in invasive ductal breast carcinoma. Hum Pathol.

[43] Fan S, Li Y, Yue P, Khuri FR, Sun SY (2010). The eIF4E/eIF4G interaction inhibitor 4EGI-1 augments TRAIL-mediated apoptosis through c-FLIP Down-regulation and DR5 induction independent of inhibition of cap-dependent protein translation. Neoplasia.

[44] Zhang X, Sai B, Wang F, Wang L, Wang Y, Zheng L (2019). Hypoxic BMSC-derived exosomal miRNAs promote metastasis of lung cancer cells via STAT3-induced EMT. Mol Cancer.

[45] Wen Q, Wang W, Chu S, Luo J, Chen L, Xie G (2015). Flot-2 expression correlates with EGFR levels and poor prognosis in surgically resected non-small cell lung cancer. PLoS ONE.

