# Supplementary material for: Exosome-mediated miR-7-5p delivery enhances the anticancer effect of Everolimus via blocking MNK/eIF4E axis in non-small cell lung cancer
Source: Cell Death Dis. 2022 Feb 8;13(2):129. doi: 10.1038/s41419-022-04565-7 (PMC8827062; doi:10.1038/s41419-022-04565-7)
Supplement: Supplementary file 1 — Supporting Information [file 41419_2022_4565_MOESM1_ESM.docx]

**Exosome-mediated miR-7-5p delivery enhances the anticancer effect of Everolimus via blocking MNK/eIF4E axis in non-small cell lung cancer**

**Supporting Information**

**Supplemental Material and Methods:**

**Reagents:** The inhibitors and antibodies were purchased from commercial sources: mTOR inhibitor Everolimus (RAD001), Mnk1 inhibitor CGP57380, exosomes inhibitor GW4869, 3-Methyladenine (3-MA) (Selleckchem, Houston, TX, USA). The synthetic miRNA probe miR-7-5p (#YD00610687) (QIAGEN, Germany), was resuspended in RNase-free water at 25 µM, probe sequence 5'-ACAACAAAATCACTAGTCTTCC-3’.

**Antibodies:** Anti-Akt antibody, anti-Mnk1 (C4C1) mAb, anti-p-Mnk1 (Thr197/202) antibody, anti-S6 (54D2) mAb, anti-p-S6 (S235/236) mAb, anti-p-S6 (S240/244) mAb, anti-eIF4E antibody, anti-4EBP1 (53H11) mAb, anti-p-4EBP1 (T37/36) mAb, anti-cleaved-PARP (Asp214) antibody, anti-DR4 (D9S1R) mAb, anti-caspase-3 (D3R6Y) mAb, anti-Bcl-xL (54H6) mAb, anti-Bak (D4E4) mAb, anti-Bax (D2E11) mAb, anti-Bad (D24A9) mAb, anti-CD9 (D3H4P) mAb, Hamartin/TSC1 (1B2) mAb (Cell Signaling Technology, MA, USA); anti-TSC2 antibody (BBI, CHINA); anti-p-Akt (S473) mAb, anti-p-eIF4E (S209) mAb, anti-CD63 antibody (Abcam, cambridge, UK); anti-GAPDH mAb, anti-α-tubulin antibody, anti-Histone-H3 antibody, anti-mTOR antibody (66888-1-IG), anti-Rab27A antibody (17817-1-AP), anti-Rab27B antibody (13412-1-AP) (Proteintech Group, CHI, USA). HRP-conjugated secondary antibody (Santa Cruz) was used as the secondary antibody.

**Cell line and cell culture****:** The human NSCLC cell lines, including A549, H157, H358, H460, H520 and SPC-A1, and immortalization of human bronchial epithelial (hBE) cells were maintained in RPMI-1640 (BI) medium supplemented with 10% fetal bovine (FBS) (BI) at 37 ℃ with 5% CO_2_. And they were recently authenticated by STR profiling and tested for mycoplasma contamination.

**Data Mining and Analysis：**LUSC data were obtained from the TCGA database (http://cancergenome.nih.gov/). A total of 496 LUSC tissues with gene expression profiles were divided into two groups named mTOR^low^ group and mTOR^high^ group according to the mean value of mTOR level in the samples, and analyzed the indicated genes enrichment between mTOR^low^ and mTOR^high^ tissues.

**MiR-7-5p target genes prediction:** We used three online analysis tools to predict miR-7-5p target genes (including Targetscan, Pictar, and TarBase), the genes that were predicted by three software at the same time were considered to be its potential target **genes.**

**Supplementary figures legend:**

**Figure S1. Everolimus targeted mTORC1 inducing NSCLC cells to secrete miR-7-5p-loaded exosomes in Rab27A and Rab27B dependent manners**

**A.** The changes of autophagy related proteins were detected by Western blotting, when NSCLC cells were treated with mTOR inhibitor 5 nM Everolimus or autophagy inhibitor 2.5 mM 3-MA alone or in combination in A549 and SPC-A1 cell lines. **B-C.** A549 cells were transfected with siTSC1/2 or siRab27A/B and treated with or without Everolimus for 24h. The western blotting detected the mTORC1, TSC1/TSC2 and Rab27A/Rab27B proteins in the corresponding cells above. **D.** The levels of miR-7-5p were measured by qPCR in miR-7-5p elevated by mimics or decreased by inhibitor in A549 cells. **E.** The level of miR-7-5p and MNK1 was confirmed by qPCR in the human bronchial epithelial (HBE) cell and other NSCLC cell lines.

**Figure S2. Exosomal miR-7-5p enhanced the anticancer effect of Everolimus in vitro.**

Indicated SPC-A1 cells were treated with or without Everolimus at the settled time. The cell proliferative ability was determined by **A.** CCK8 assay and **B.** cell clone-formation assay. The cell migration and invasive ability was determined by **C.** cell scratch test and **D.** transwell matrigel assay. SPC-A1 cells were treated with indicated exosomes or Everolimus for settled times. The cell proliferative ability was determined by **E.** CCK8 assay and **F.** cell clone-formation assay. The cell migrating and invasive ability was determined by **G.** cell scratch test and **H.** transwell matrigel assay. Data are shown as mean ± SD. **P* < 0.05, ***P* < 0.01, and ****P*< 0.001 compared with control.

**Figure S3. Exosomal miR-7-5p enhanced the anticancer therapeutic efficacy of Everolimus in vivo**

**A-B.** The growth rate of xenograft tumors in nude mouse subcutaneous tumor models. **C.** The quantification of the immunohistochemical/ *in situ* hybridization staining for Ki67, MNK1, p-eIF4E^S209^ and miR-7-5p in the tumor tissues. **D.** Luminescence signals of intraperitoneal A549-Luc tumor xenografts from different treatment groups at the indicated week.

**Figure S4. Combination of miR-7-5p with Everolimus induced apoptosis to exhibit a synergistic anticancer therapeutic efficacy via dual abrogation of MNK/eIF4E and mTOR in NSCLC.**

Analysis of proteins of p-MNK^Thr197/202^ and p-eIF4E^S209^ respectively extracted from cytosolic or nuclear in indicated treatment of SPC-A1 cells was examined by Western blotting. **A.** SPC-A1 cells were treated with MNK1 inhibitor CGP57380 or mTOR inhibitor Everolimus alone or in combination. **B.** The LV-miR-7-5p or LV-NC SPC-A1 cells were treated with or without Everolimus. **C.** Treatment of SPC-A1 cells with Everolimus or miR-7-5p loaded exosomes alone or in combination. **D.** Cells were transfected with indicated mimics with or without Everolimus for 48 h. Apoptotic cells were detected by flow cytometry using Annexin V/PI staining. Columns, means of three replicate determinations; each bar represents mean ± SD. NS, non-significant, **P*<0.05, ** *P*<0.01. **E-G**. The stably LV-miR-7-5p SPC-A1 cells were treated with Everolimus or DMSO for 48 h. Cell lysates were harvested to detect the indicated apoptotic proteins by western blotting analysis and GAPDH was used as a loading control.
