# Supplementary figures and images for: Exosome-mediated miR-7-5p delivery enhances the anticancer effect of Everolimus via blocking MNK/eIF4E axis in non-small cell lung cancer
Source: Cell Death Dis. 2022 Feb 8;13(2):129. doi: 10.1038/s41419-022-04565-7 (PMC8827062; doi:10.1038/s41419-022-04565-7)

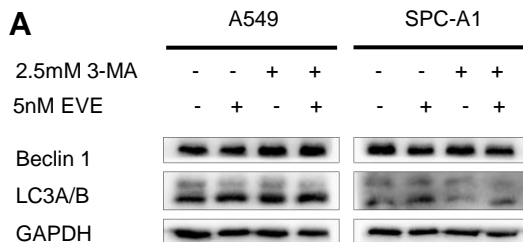

**B**

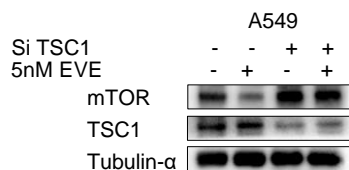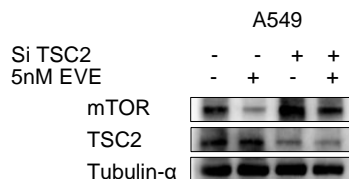

**C**

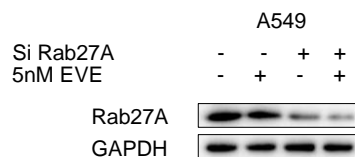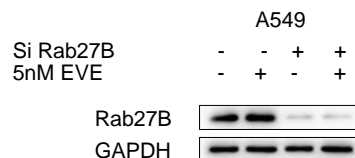

**D**

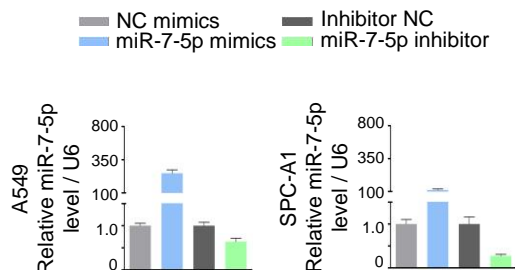

**E**

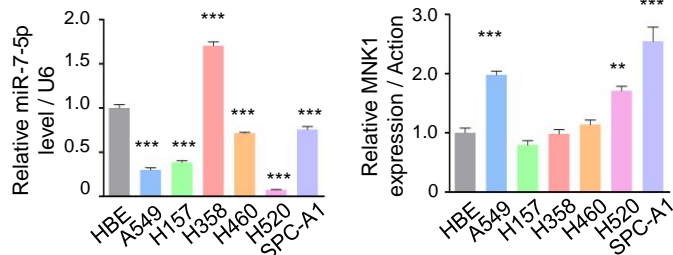

Supplement: Supplementary file 2 — Figure S1. [file 41419_2022_4565_MOESM2_ESM.pdf]

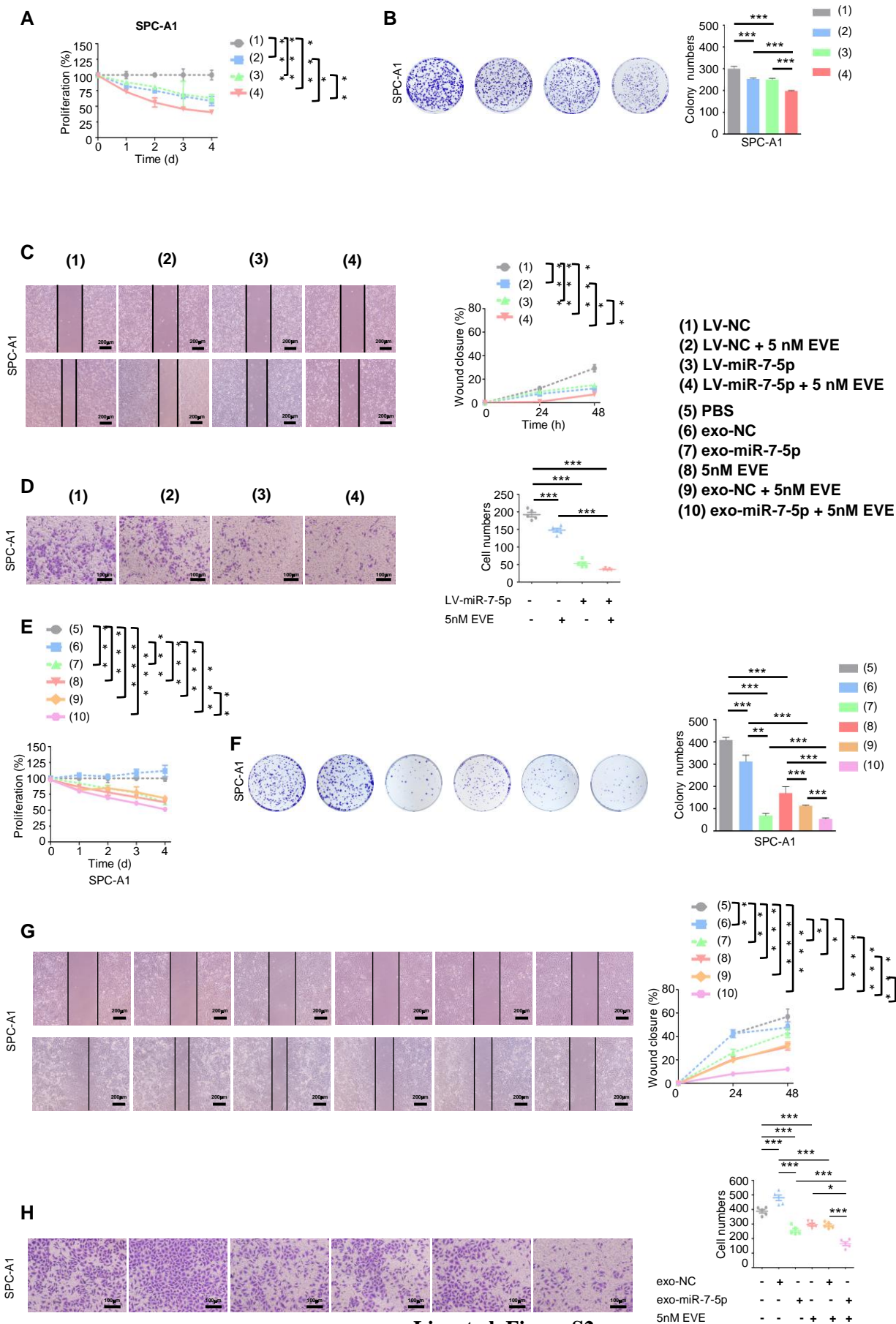

Liu, et al. Figure S2

Supplement: Supplementary file 3 — Figure S2. [file 41419_2022_4565_MOESM3_ESM.pdf]

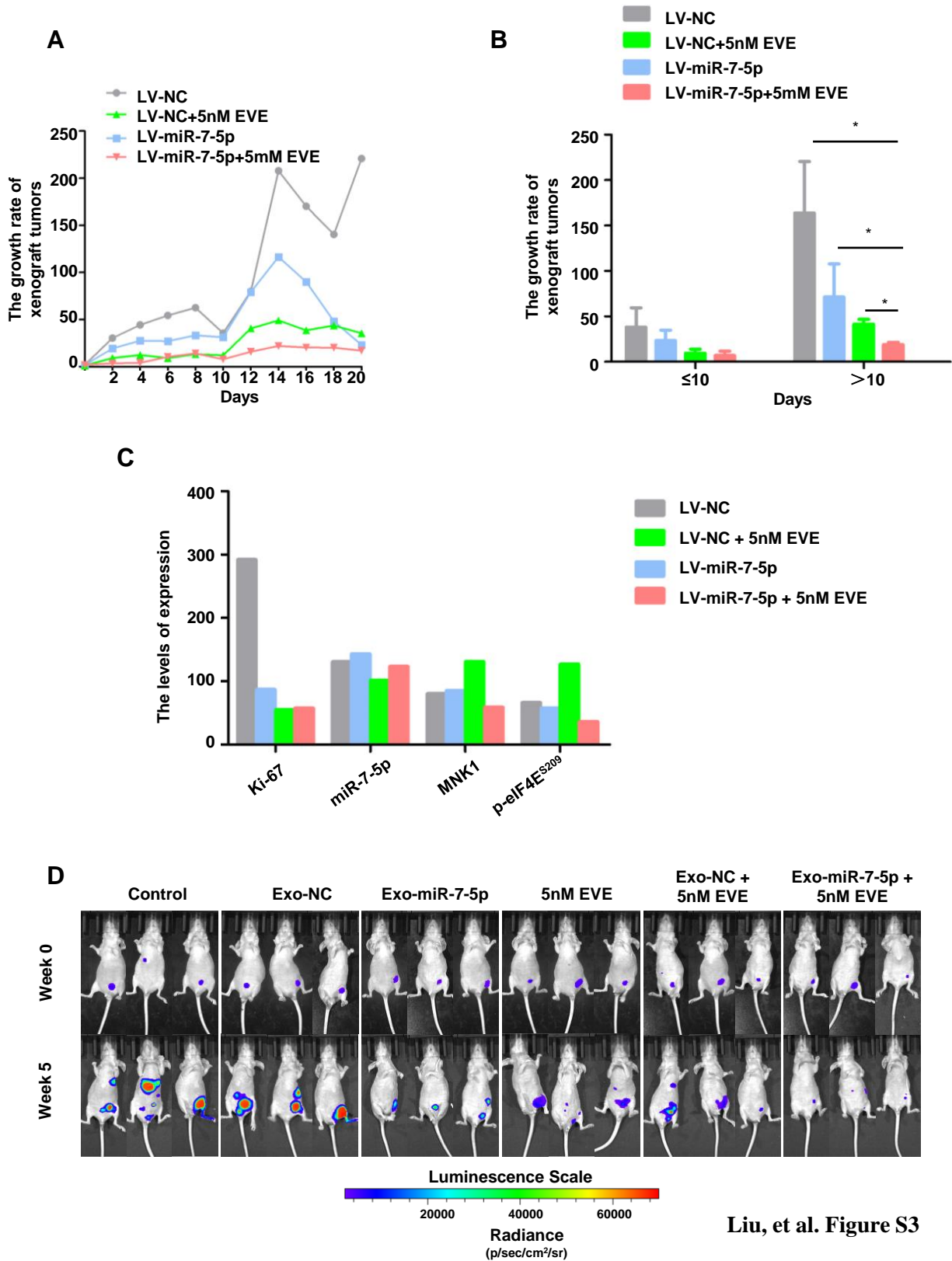

Supplement: Supplementary file 4 — Figure S3. [file 41419_2022_4565_MOESM4_ESM.pdf]

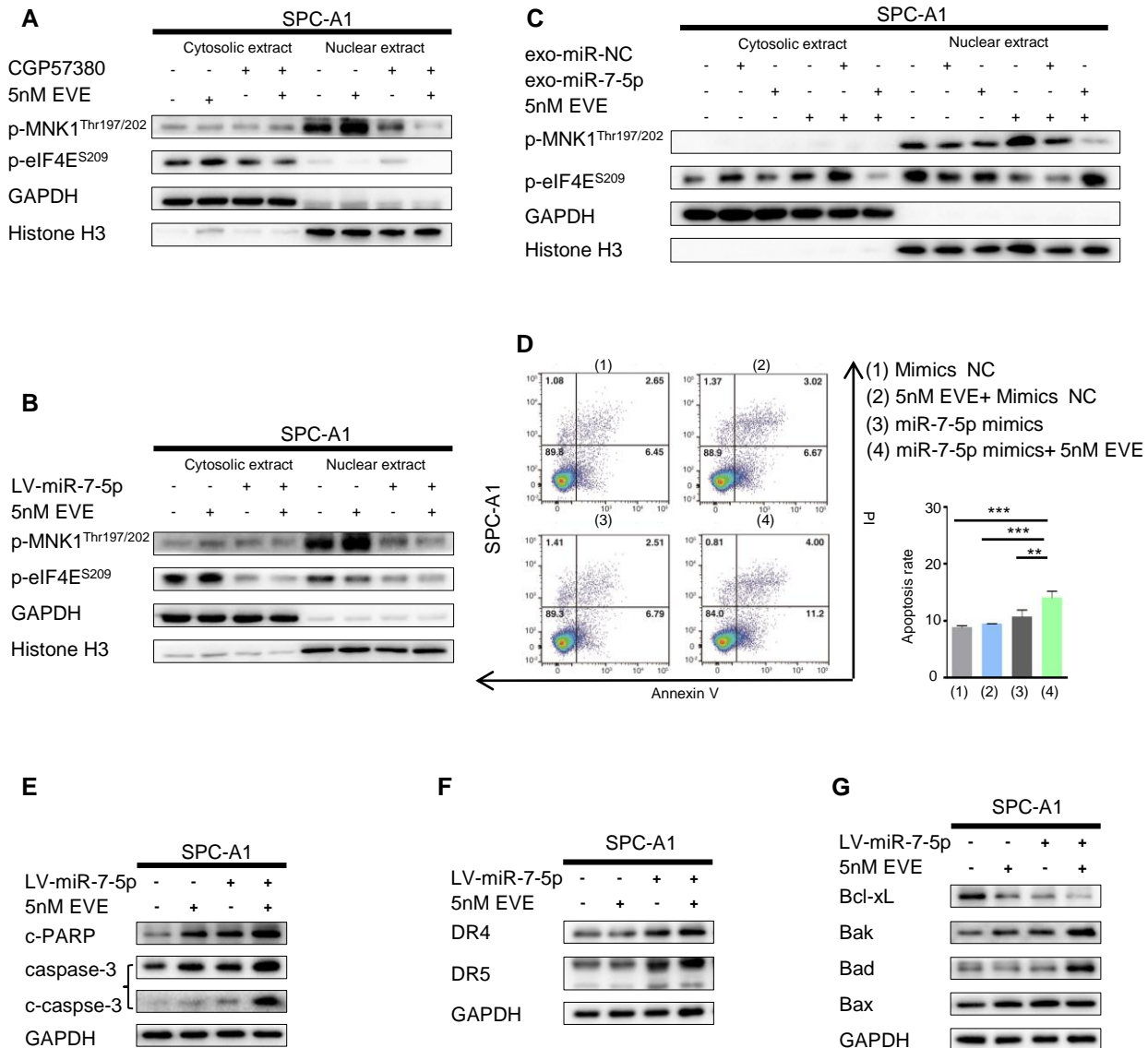

Liu, et al. Figure S4

Supplement: Supplementary file 5 — Figure S4. [file 41419_2022_4565_MOESM5_ESM.pdf]
