# Supplementary material for: Exosome-mediated miR-7-5p delivery enhances the anticancer effect of Everolimus via blocking MNK/eIF4E axis in non-small cell lung cancer
Source: Cell Death Dis. 2022 Feb 8;13(2):129. doi: 10.1038/s41419-022-04565-7 (PMC8827062; doi:10.1038/s41419-022-04565-7)
Supplement: Supplementary file 6 — Table S1. [file 41419_2022_4565_MOESM6_ESM.docx]

**Table S1. The potential targets of miR-7-5p predicted by three online analysis tools**

| **Gene symnol** | **Targetscan**  **(Total context++ score)** | **Tarbase (Pred. score)** | **PicTar (score)** |
| --- | --- | --- | --- |
| *RNF141* | -0.55 | 0.995 | 1.48 |
| *SEMA4C* | -0.23 | 0.911 | 6.85 |
| *POLE4* | -1.45 | 1 | 8.56 |
| *CNOT8* | -0.61 | - | 3.87 |
| *NXT2* | -0.9 | - | 4.77 |
| *RAF1* | -0.86 | - | 4.03 |
| *MAP3K9* | -0.2 | - | 3.8 |
| *SPATA2* | -0.95 | - | 15.72 |
| *VDAC1* | -0.56 | - | 2.63 |
| *LEMD3* | -0.54 | 0.856 | 2.63 |
| *KLF4* | -0.58 | - | 8.44 |
| *CNN3* | -0.53 | - | 2.74 |
| *ZNF395* | -0.39 | - | 4.36 |
| *PLXNA1* | -0.13 | - | 3.95 |
| *RSBN1* | -0.37 | 0.953 | 5.51 |
| *CKAP4* | -0.45 | - | 2.3 |
| *PFN2* | -0.45 | - | 2.26 |
| *POGK* | -0.27 | - | 1.61 |
| *SMARCD1* | -0.52 | 0.851 | 6.67 |
| *SP1* | -0.79 | - | 6.14 |
| *SOCS2* | -0.1 | - | 2.36 |
| *BACE1* | -0.12 | - | 3.14 |
| *KPNA1* | -0.24 | 0.977 | 1.75 |
| *PAPPA* | -0.01 | 0.906 | 2.71 |
| *OSBPL11* | -0.28 | - | 2.06 |
| *CALU* | -0.23 | - | 1.53 |
| *ARF4* | -0.41 | - | 2.98 |
| *TCF12* | -0.44 | 0.916 | 1.56 |
| *DDIT4* | -0.31 | 0.915 | 3.41 |
| *PSME3* | -0.76 | - | 5.95 |
| *GLTSCR1* | -0.29 | 0.969 | 5.74 |
| *SMYD5* | -0.36 | - | 2.14 |
| *EIF4EBP2* | -0.66 | - | 3.6 |
| *SERP1* | -0.39 | - | 3.79 |
| *MKNK1* | -0.31 | - | 4.52 |
| *OGT* | -0.16 | - | 1.84 |
