# Supplementary material for: Exosome-mediated miR-7-5p delivery enhances the anticancer effect of Everolimus via blocking MNK/eIF4E axis in non-small cell lung cancer
Source: Cell Death Dis. 2022 Feb 8;13(2):129. doi: 10.1038/s41419-022-04565-7 (PMC8827062; doi:10.1038/s41419-022-04565-7)
Supplement: Supplementary file 7 — Table S2. [file 41419_2022_4565_MOESM7_ESM.docx]

**Table S2. Primers and sequences used in this study**

| **Gene symnol** | **Primer sequence** |
| --- | --- |
| miR-7-5p | Forward: TGGAAGACTAGTGATTTTGTTGTT |
| cel-miR-39-3p | Forward: TCACCGGGTGTAAATCAGCTTG |
| eIF4E | Forward: ATGTGGCGCTGTTGTTAATGT |
|  | Reverse: CTGCGTGGGACTGATAACCAA |
| MKNK1 | Forward: AGATGGGCAGTAGCGAACC |
|  | Reverse: AGCAATTCAGAGGTCAGCTTG |
| Actin | Forward: GAGCTACGAGCTGCCTGACG |
|  | Reverse: GTAGTTTCGTGGATGCCACAG |

A universal primer against the stem-loop region and U6 primers Provided by Mir-X miRNA First-Strand synthesis kit (Takara).
