# Supplementary material for: Exosome-mediated miR-7-5p delivery enhances the anticancer effect of Everolimus via blocking MNK/eIF4E axis in non-small cell lung cancer
Source: Cell Death Dis. 2022 Feb 8;13(2):129. doi: 10.1038/s41419-022-04565-7 (PMC8827062; doi:10.1038/s41419-022-04565-7)
Supplement: Supplementary file 8 — Table S3. [file 41419_2022_4565_MOESM8_ESM.docx]

**Table S3. siRNA sequences used in this study**

|  | **sequence** |
| --- | --- |
| si TSC1 | sense: GCACUCUUUCAUCGCCUUUTT |
|  | antisense: AAAGGCGAUGAAAGAGUGCTT |
| si TSC2 | sence: GCAUGGAAUGUGGCCUCAATT |
|  | antisense: UUGAGGCCACAUUCCAUGCTT |
| si Rab27A | sence: GTGGGCATTGATTTCAGGGAA |
|  | antisense: TTCCCTGAAATCAATGCCCAC |
| si Rab27B | sence: TAGGAATAGACTTTCGGGAAA |
|  | antisense: TTTCCCGAAAGTCTATTCCTA |
