# Supplementary material for: Exosome-mediated miR-7-5p delivery enhances the anticancer effect of Everolimus via blocking MNK/eIF4E axis in non-small cell lung cancer
Source: Cell Death Dis. 2022 Feb 8;13(2):129. doi: 10.1038/s41419-022-04565-7 (PMC8827062; doi:10.1038/s41419-022-04565-7)
Supplement: Supplementary file 9 — Table S4. [file 41419_2022_4565_MOESM9_ESM.docx]

**Table S4. mimics and inhibitors sequences used in this study**

|  | **sequence** |
| --- | --- |
| miR-7-5p mimics | sense: UGGAAGACUAGUGAUUUUGUUGUU |
|  | antisense: CAACAAAAUCACUAGUCUUCCAUU |
| NC mimics | sence: UUCUCCGAACGUGUCACGUTT |
|  | antisense: ACGUGACACGUUCGGAGAATT |
| miR-7-5p inhibitor | sence: ATGTGGCGCTGTTGTTAATGT |
| Inhibitor NC | sence: CAGUACUUUUGUGUAGUACAA |
