# Supplementary material for: Exosome-mediated miR-7-5p delivery enhances the anticancer effect of Everolimus via blocking MNK/eIF4E axis in non-small cell lung cancer
Source: Cell Death Dis. 2022 Feb 8;13(2):129. doi: 10.1038/s41419-022-04565-7 (PMC8827062; doi:10.1038/s41419-022-04565-7)
Supplement: Supplementary file 10 — Table S5. [file 41419_2022_4565_MOESM10_ESM.docx]

**Table S5. Lentivirus sequences used in this study**

|  | **sequence** |
| --- | --- |
| LV3-NC | sence: TTCTCCGAACGTGTCACGT |
| LV3-hsa-miR-7-5p mimics | sence: TGGAAGACTAGTGATTTTGTTGTT |
